# The definitions of three‐dimensional landmarks on the human face: an interdisciplinary view

**DOI:** 10.1111/joa.12407

**Published:** 2015-12-11

**Authors:** Stanislav Katina, Kathryn McNeil, Ashraf Ayoub, Brendan Guilfoyle, Balvinder Khambay, Paul Siebert, Federico Sukno, Mario Rojas, Liberty Vittert, John Waddington, Paul F. Whelan, Adrian W. Bowman

**Affiliations:** ^1^ Institute of Mathematics and StatisticsMasaryk UniversityBrnoCzech Republic; ^2^ School of Mathematics and StatisticsThe University of GlasgowGlasgowUK; ^3^ College of MVLSSchool of MedicineDental SchoolThe University of GlasgowGlasgowUK; ^4^ Institute of Technology, TraleeKerryIreland; ^5^ School of DentistryThe University of LeedsLeedsUK; ^6^ School of Computing ScienceThe University of GlasgowGlasgowUK; ^7^ Department of Information and Communication TechnologiesPompeu Fabra UniversityBarcelonaSpain; ^8^ Molecular and Cellular TherapeuticsRoyal College of Surgeons in IrelandDublinIreland; ^9^ Centre for Image Processing and AnalysisDublin City UniversityDublinIreland

**Keywords:** curves, landmarks, reproducibility, shape

## Abstract

The analysis of shape is a key part of anatomical research and in the large majority of cases landmarks provide a standard starting point. However, while the technology of image capture has developed rapidly and in particular three‐dimensional imaging is widely available, the definitions of anatomical landmarks remain rooted in their two‐dimensional origins. In the important case of the human face, standard definitions often require careful orientation of the subject. This paper considers the definitions of facial landmarks from an interdisciplinary perspective, including biological and clinical motivations, issues associated with imaging and subsequent analysis, and the mathematical definition of surface shape using differential geometry. This last perspective provides a route to definitions of landmarks based on surface curvature, often making use of ridge and valley curves, which is genuinely three‐dimensional and is independent of orientation. Specific definitions based on curvature are proposed. These are evaluated, along with traditional definitions, in a study that uses a hierarchical (random effects) model to estimate the error variation that is present at several different levels within the image capture process. The estimates of variation at these different levels are of interest in their own right but, in addition, evidence is provided that variation is reduced at the observer level when the new landmark definitions are used.

## Introduction

Biological shape is a topic of considerable scientific interest that has a very long history and that has many applications across the range of biological species. In terms of measurement, anatomical landmarks have been the basis of quantitative assessment and modelling of shape since the pioneering work of Thompson (1963, originally published in 1917) and Martin & Saller (1957). The identification of points that are well‐defined and have anatomical meaning allows shape to be characterised in a manner that corresponds across subjects and that therefore provides the basis of subsequent statistical analysis. Bookstein (1991) and Dryden & Mardia (1998) give detailed descriptions of a very substantial body of methods that are now available for routine use and that have had an enormous influence in anthropological and medical studies from many different application areas. In particular, these methods allow a proper analysis of shape, expressed in the complete three‐dimensional configuration of landmarks, rather than reducing the information to particular distances and angles between selected landmarks. Shape is broadly defined as the information that remains after location, orientation, and possibly also scale, have been removed.

For the important application area of the human face, detailed definitions of landmarks were provided by Farkas (1994) and this remains a standard reference. However, many of these definitions are given in terms of two‐dimensional views. This was entirely appropriate when visual inspection, or two‐dimensional photographic or X‐ray images, provided the starting point, but there are significant difficulties associated with this approach. A major issue is the requirement to place the head in an orientation that gives well‐defined meaning to, and reproducible identification of, the landmarks of interest. A common approach is to use the Frankfurt horizontal plane, which is defined in terms of three anatomical landmarks: left orbitale and left and right porion. The left orbitale, lies at the lowest points of the eye sockets, but this is defined by bone and so estimation is made more difficult by the soft tissue that is superimposed. The left and right porion lie at the upper perpendicular projection onto the soft tissue at the external end of the ear canal. All these locations require careful training to identify, and however well this is done some error is inevitable. The use of the left, but not right, orbitale immediately creates difficulties as a result of asymmetry. This could be addressed by the use of both orbitale but since a plane requires only three points for mathematical definition, the use of four must necessarily involve some compromise. The difficulty is that quite a number of other important landmarks, for example on the nose tip and chin, then take their definitions from this planar reference. In addition, a further vertical planar reference is required and the mid‐sagittal plane is commonly used for this. The definition of this plane is based on the landmarks that lie on the mid‐line of the face, which again leads to the difficulty of estimating landmarks before this plane is in place, as well as compromising across multiple landmarks.

Weber & Bookstein (2011) address some of these issues by taking a three‐dimensional perspective in the context of the skull, where landmarks can be categorised by their relationship to broader features of the skull. For example, some landmarks lie at the crossing point of three‐dimensional curves, such as the intersection of a ridge curve with the mid‐line curve lying in the mid‐sagittal plane. This type of thinking clearly extends to the face, although the pliable nature of soft tissue makes identification in practice more problematic.

The aim of the present paper is to propose new definitions of facial landmarks that are not dependent on the orientation of the head. These are based on three‐dimensional surface characteristics and the key information used to characterise landmarks is curvature. This refers in general to the local shape of the facial surface but, following Weber & Bookstein (2011), anatomical curves across the face can also often provide key characteristics that inform landmark definitions.

The approach is interdisciplinary, providing insights from biology, clinical use, computer vision, differential geometry and statistical analysis. Good landmark definitions require the relevant information to be readily identifiable, with good intra‐ and inter‐person reproducibility. These also need to apply to different imaging modalities such as stereophotogrammetry and laser scanning.

In Biological and clinical perspectives, the manner in which the scientific and clinical questions underlying the need for data collection and analysis inform the process of definition is discussed. The issues associated with imaging technology and computer vision are discussed in Imaging perspective. This leads in A geometrical perspective to a discussion of curvature from the perspective of differential geometry and to specific new definitions of anatomical landmarks on the face. A study of reproducibility of landmark identification based on these definitions is reported in Validation study where the variations present at multiple levels of the imaging process are identified and where some evidence of reduced inter‐observer variation with the new landmark definitions is apparent. Some final discussion is given in Discussion.

## Biological and clinical perspectives

There are many reasons why the study of human facial shape is of interest. Two particular examples, involving issues of biological development and the need to assess the outcome of facial surgery, are described below in order to highlight the wide range of motivations for interest in facial shape and to identify issues that can inform the selection, definition and identification of anatomical landmarks.

### The origins of schizophrenia

In the early embryological stages of human development, the anterior brain and the face are very closely connected. As a result, disorders of early brain development can be associated with facial dysmorphology. The origins of schizophrenia provide a particular example. A specific study is described by Hennessy et al. (2007), who used three‐dimensional laser surface imaging to capture the facial surface of patients who satisfied DSM‐IV criteria for schizophrenia, in comparison with control subjects. Twenty‐six anatomical landmarks were manually identified on each facial surface image. Interpolation using thin‐plate splines was then used to generate a larger number of surface points (semi‐landmarks) for analysis. The aim was to identify aspects of facial shape that distinguish schizophrenia patients from controls, and both male and particularly female patients showed evidence of significant facial dysmorphology. This included a narrowing and reduction of the mid/lower face and fronto‐nasal prominences, with reduced width and posterior displacement of the mouth, lips and chin in particular. There was also evidence of increased width of the upper face, mandible and skull base, with lateral displacement of the cheeks, eyes and orbits, and anterior displacement of the superior margins of the orbits. The conclusion was that the fronto‐nasal prominence has a characteristic topography that is associated with schizophrenia. The fact that this is precisely the area of the face that has the closest embryological relationship with the anterior brain points to possible common early developmental perturbation. In a related study, Prasad et al. (2015) analyse the facial dysmorphology associated with 22q11.2 deletion syndrome (velocardiofacial syndrome, associated with high risk for psychosis) and compare this with that of schizophrenia.

### Assessing the outcome of facial surgery

In a very different setting, facial surgery aims to alter an existing facial shape, usually to address the effects of trauma or of congenital issues. For example, cleft‐lip and/or palate is one of the commonest forms of facial disfigurement in children, and there have been a variety of initiatives to assess the effectiveness of surgical repair. This includes the study described by Hood et al. (2003) where the facial shapes of 3‐month‐old infants exhibiting unilateral clefts and non‐cleft controls of the same age were compared. Images of these children were also captured at 6 months and 1 year old, after surgical correction of the cleft cases. Particular interest lay in the degree of facial asymmetry, quantified by the mismatch between manually identified anatomical landmarks and their reflections, after Procrustes registration, as described by Bock & Bowman (2006). Control asymmetry scores showed no evidence of change across time, while the cleft groups displayed immediate reduction after surgery, followed by moderate improvement over time.

These examples underline the central role of anatomical landmarks in quantifying shape in general and important derived measures such as asymmetry in particular. The accuracy and reproducibility of landmarks is therefore of high importance as variation here will be transmitted to subsequent analysis. This strengthens the need to reconsider the definitions of landmarks, to overcome the difficulties associated with two‐dimensional perspectives and to take advantage of the direct three‐dimensional surface information available.

## Imaging perspective

The relatively recent advent of three‐dimensional surface imaging methods provides rich and complete representations of surface shape. However, the accuracy of subsequent landmarking will clearly be highly influenced by the accuracy of the underlying surface. Some approaches to three‐dimensional imaging are outlined here, with particular focus on the accuracy that can be achieved.

Stereo‐camera systems capture the image of an object from two or more viewpoints in a synchronised manner. The main advantages of this type of system are the short capture time and the wide range of environments in which they can operate. The three‐dimensional reconstruction is performed offline at a high computational cost but with the availability of high‐quality cameras this approach can reach very high accuracy, up to 0.1 mm. Structured light approaches project two‐dimensional patterns of light onto the object of interest and this can achieve high accuracy, up to 0.3 mm, but these systems are sensitive to lighting conditions. Boehnen & Flynn (2005) and Al‐Khatib (2010) document the details of these and other approaches.

Laser methods project a beam of light (spot or stripe) onto the object of interest and extract three‐dimensional information by triangulation from the image captured by a camera. Convenient, hand‐held versions of this technology are now available. This approach is characterised by low computational cost, longer capture time and size limitation, but the accuracy achieved is very high, up to 0.05 mm (Boehnen & Flynn, 2005; Sansoni et al. 2009).

The Shape from *X* approaches, where *X* may refer to Texture, Defocus or Shading, use only one view from a single camera. However, the three‐dimensional surface has to be inferred through information on orientation and so these approaches are not well suited for applications requiring high accuracy and resolution (Moons et al. 2009; Sansoni et al. 2009; Pears et al. 2012).

Beyond the intrinsic accuracy of the imaging system, there are many other levels at which variation may be present. These include the facial expression of the subject, the lighting conditions and other environmental factors of the image capture session that may affect the quality of the reconstruction. It is important to develop a good protocol and adhere to this during image capture so that the effects of these different sources of variation can be contained as far as possible. However, even where high‐quality images have been captured there are additional issues associated with the subsequent identification of anatomical landmarks. In manual identification, the human visual perception system plays a central role. While this system is highly attuned to some aspects of shape, the accurate location of individual points is a less common task for which training and experience is required. A statistical model to assess the size of the variabilities in landmark identification, at all these different levels, is discussed in the context of a validation study described in Validation study. However, as discussed briefly in the Introduction above, clear and unambiguous definitions are an essential starting point, and new proposals for these are made in the following section.

## A geometrical perspective

The critical importance of landmarks in providing key information on three‐dimensional shape was emphasised in Biological and clinical perspectives, while the difficulties associated with standard definitions of landmarks, involving careful orientation and two‐dimensional perspectives, was highlighted in the Introduction. A potential solution is available through the mathematical characterisation of local surface shape. This provides quantifiable measures of shape that are fully three‐dimensional and independent of orientation. The details of this approach, and consequent proposals for new definitions of landmarks, are developed in this section.

The classical geometry of surfaces is well understood. Koenderink (1990) gives a wide‐ranging and thorough introduction, while Koenderink & van Doorn (1992) provide a very accessible summary of the main ideas, including those sketched below. The key concept is that the local shape at almost all points on a surface can be represented in the form

$$z=\frac{1}{2}\left(\kappa_{1}x^{2}+\kappa_{2}y^{2}\right),$$

where *z* lies in the ‘normal’ (perpendicular) direction to the surface and the orthogonal axes *x* and *y* lie on the ‘tangent plane’. The axes *x* and *y* correspond to the directions of maximum and minimum curvature (rate of bending) of the surface. The values of these maximum and minimum curvatures, known as principal curvatures are $\kappa_{1}$, $\kappa_{2}$, where by convention $\kappa_{1}\geq \kappa_{2}$. Note that the axes *x*,* y* and *z* are defined locally around the current surface point of interest and these axes will change as the point of interest is moved.

**Figure 1:**
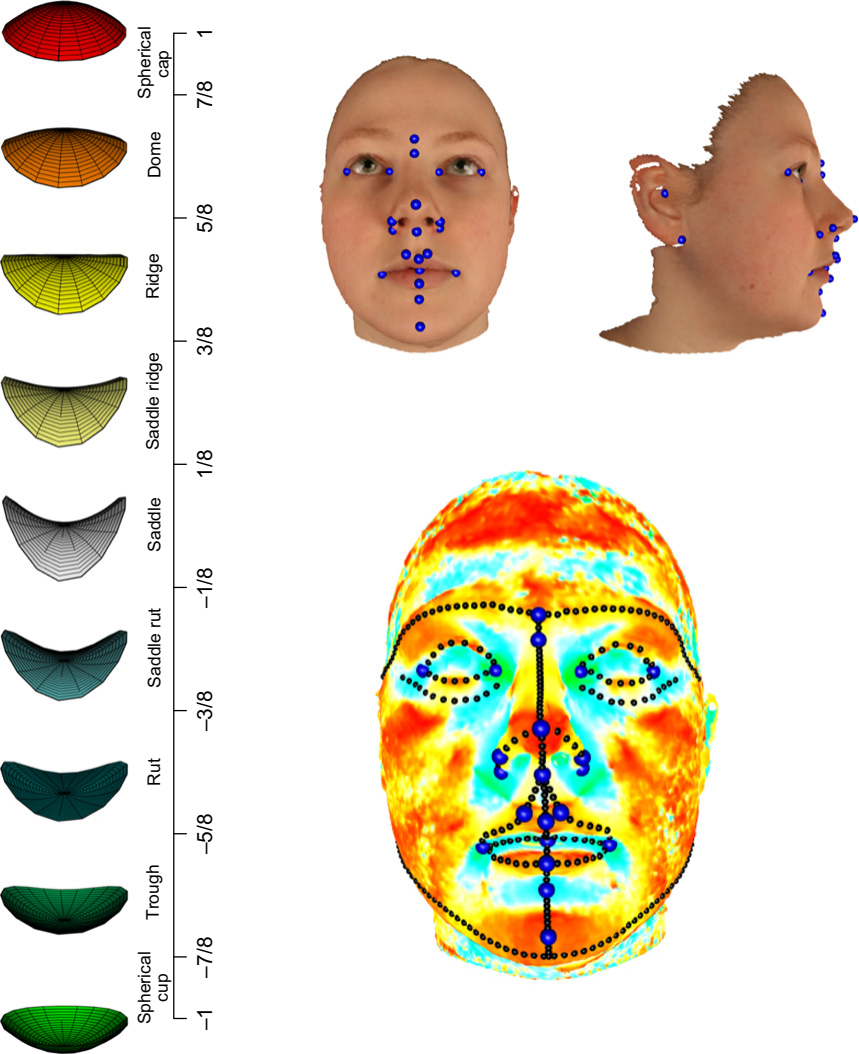
The left‐hand column of plots shows the local surfaces associated with the shape index on the scale from −1 to 1, with colour coding to identify each shape category. The top two facial images show the location of manually identified landmarks. The bottom images are coloured by the value of the shape index, and have the landmarks and anatomical curves superimposed.

It is the principal curvatures, which are independent of the location and orientation of the surface, which provide the essential characterisation of local shape. In particular, a shape index can be defined as

$$S=\frac{2}{\pi }{\text{tan}}^{-1}\left(\frac{\kappa_{2}+\kappa_{1}}{\kappa_{2}-\kappa_{1}}\right),$$

where $\tan^{-1}$ denotes the inverse tan function. The purpose of this index is to characterise local surface shape in a systematic and interpretable manner. Values of S close to −1 indicate a ‘spherical cup’ shape where the curvatures $\kappa_{1}$ and $\kappa_{2}$ are positive and very close to one another. As S increases, the corresponding surfaces bend smoothly through ‘trough’ and ‘rut’ shapes, towards a ‘saddle’ that is most pronounced at S=0. As S increases through the positive half of the scale, this process is reversed until a ‘spherical cap’ is produced. This is the converse of the original ‘spherical cup’, now with negative rather than positive values of the principal curvatures $\kappa_{1}$ and $\kappa_{2}$. Koenderink & van Doorn (1992) describe the types of shape that are exhibited along this continuum, together with the corresponding ranges of S, appropriate verbal descriptors and associated colour codes. These are shown in the left hand side Fig. 1, which is modelled on Fig. 5 of Koenderink & van Doorn (1992). An appealing feature of the shape index is that it is influenced only by the relative sizes of $\kappa_{1}$ and $\kappa_{2}$. If each curvature is multiplied by the same constant then the shape index remains unaltered. This reflects the fact that the intrinsic shape of each feature, such as a spherical cup for example, is unchanged by whether this cup is shallow or deep.

The upper facial images in Fig. 1 show the locations of a set of traditional anatomical landmarks, while the lower image is coloured by the value of the shape index, constructed by the simple device of fitting a quadratic surface to the surface points within a 1 cm radius of each surface location. It is clear that these colours track the major features of the face, such as the caps and domes of the nose tip and chin, the ruts of the mouth and around the nose and eyes, and the ridges of the nose and lips. (The stippled patterns across the cheeks and forehead are artefacts of the reconstruction algorithm that sometimes occur on flat surfaces.) An appealing approach to defining landmarks is therefore to characterise them by their location with respect to these large‐scale features. Specifically, this requires the identification of a set of anatomical curves which track the ridges and ruts of the facial surface. Landmarks can then be characterised as locations where these curves bend most strongly, such as alare crest where the ridge along the alar section of the nose meets the paranasal area, or where two curves cross, such as endocanthion where the upper and lower eyelid curves meet. The use of anatomical curves is helpful as an intermediary step as it is easier to identify the pattern of a large‐scale feature than to focus immediately on a single point location.

This approach requires the definition of a set of anatomical curves across the face. Proposed definitions are given in Table 1 and displayed on the lower facial image in Fig. 1. In interpreting these definitions, it is helpful first to give some explanation of how curves can be characterised from the values of the surface shape index. In practice, most observers find it relatively straightforward to locate the line of a ridge on a surface, but a precise mathematical definition requires a little care. Informally, a ridge is defined as a continuous set of points, each of which has a shape index appropriate to a ridge point and which locally has a stronger ridge shape index than neighbouring values that are at right angles to the direction of the ridge at that location. A rut has a similar definition, using a different section of the shape index scale. Ridges and ruts are the most common features of interest, but sometimes these disappear before reaching the end points of interest. For example, this can happen with the ridges of the philtrum, in which case it is convenient to define the continuation of a curve. This can be characterised as the set of points that follow on continuously across the surface, either in the same direction as the last identifiable direction of the curve or towards some other location of interest or to the closest point on another curve. An example is the brow ridge that is extended in Fig. 1 towards tragus in the ear. (Curves on the ears have not been displayed on Fig. 1 because this region is often subject to inaccuracy in frontal imaging systems and anatomical interest is most often directed towards the central regions of the face. When the ears are of interest, it may be more advisable to target the imaging of this region directly.)

**Table 1:** Definitions of anatomical curves

	Anatomical curves
Brow ridge	Ridge points at the supra‐orbital region of the forehead
Inferior orbital	Rut points immediately below the lower eyelids
Lower/upper eye lid	The superior and inferior edges of the palpebral fissure
Alar	Ridge points on the lateral extension of the nasal cartilage
Philtrum ridge	Ridge points immediately lateral to the mid‐line philtrum
Labial seal	Rut points where the upper and lower lips meet
Lower/upper lip	Ridge points along the lower/upper lip
Ear rim	Ridge points on the peripheral boundary of the ear cartilage, constituting the helix and the ear lobe
Tragus	Ridge points on the rim of the tragus, terminating with the superior and inferior points of maximum curvature at the margins of the tragus
Mandible	Ridge points across the entire mandible (lower jaw)
Mid‐line nasal profile	Ridge points from the nasal root along the dorsum of the nose and the columella
Mid‐line philtrum	Rut points between the columella and the upper lip
Mid‐line upper lip	The continuation of the philtrum curve to the closest point on the labial seal curve
Mid‐line lower lip	The continuation of the mid‐line upper lip curve to the closest point on the lower lip curve
Mid‐line mentolabial	The continuation of the mid‐line lower lip curve to the closest point of the mentolabial sulcus (rut)
Mid‐line chin	The continuation of the mid‐line mentolabial curve to the closest point on the mandible curve

In the subsequent definition of landmarks, the crossing of two curves is a simple concept, but the idea of the curvature of a curve, referred to as geodesic curvature, merits a little more consideration. An intuitive characterisation is simply how quickly a curve bends at each point on its path. However, for the record, this can be given a precise definition as

(1) $$\kappa \left(s\right)=\frac{\sqrt{{\left(z{}^{\prime }{}^{\prime }y{}^{\prime }-y{}^{\prime }{}^{\prime }z{}^{\prime }\right)}^{2}+{\left(x{}^{\prime }{}^{\prime }z{}^{\prime }-z{}^{\prime }{}^{\prime }x{}^{\prime }\right)}^{2}+{\left(y{}^{\prime }{}^{\prime }x{}^{\prime }-x{}^{\prime }{}^{\prime }y{}^{\prime }\right)}^{2}}}{{\left(x{{}^{\prime }}^{2}+y{{}^{\prime }}^{2}+z{{}^{\prime }}^{2}\right)}^{\frac{3}{2}}},$$

where *x*,* y*,* z* describe how the co‐ordinates of the curve change as we travel along it. Specifically, *x*,* y*,* z* are functions of *s* that represent the distance of a particular point along the curve from start to finish. The quantities $x{}^{\prime }$, $y{}^{\prime }$, $z{}^{\prime }$, $x{}^{\prime }{}^{\prime }$, $y{}^{\prime }{}^{\prime }$, $z{}^{\prime }{}^{\prime }$ denote the first and second derivatives of these functions (see Koenderink, 1990 for details).

Tables 2 and 3 below propose new definitions of anatomical landmarks based on points of maximum curvature along curves and on the crossing of curves. In the tables, each landmark has two definitions; the first (in italics) is the traditional one, following Farkas (1994), while the second (in normal font) is the new proposal. For ease of reference, the landmarks are ordered in a superior–inferior direction.

**Table 2:** Landmarks defined on single curves. In each case, traditional definitions are given in italics and the new definitions in normal font

	Landmarks on single curves
Sellion	*The most posterior point of the frontonasal soft tissue contour in the midline of the base of the nasal root*
	The point of maximal curvature of the mid‐line nasal profile curve at its nasal root end
Subnasale	*The point where the nasal septum merges with the upper cutaneous lip in the mid‐sagittal plane*
	The point of maximal curvature on the mid‐line curve at the base of the nasal septum
Alare	*The most lateral point on each alar contour*
	The point of maximal curvature along the alar curve
Alare crest	*The facial insertion of each alar base*
	The point of maximum curvature on the alar curve where this meets the paranasal area
Cheilion	*The point located at each labial commissure*
	The point of maximum curvature at the lateral end of the labial seal curve
Sublabiale	*The most posterior mid‐point on the labiomental soft tissue contour that defines the border between the lower lip and the chin*
	The point of maximal curvature in the mid‐line curve as it passes through the *mentolabial sulcus*
Tragion	*The point located at the upper margin of each tragus*
	The point of maximum curvature at the superior end of the tragus curve
Otobasion inferius	*The point of attachment of the ear lobe to the cheek, which determines the lower border of the ear insertion*
	The final point at the preauricular end of the ear rim curve.

**Table 3:** Landmarks defined by the crossing of two curves. In each case, traditional definitions are given in italics and the new definitions in normal font

	Landmarks at the crossing of two curves
Exocanthion	*The soft tissue point located at the outer commissure of each eye fissure*
	The crossing of the lateral ends of the lower and upper eye lid curves
Endocanthion	*The soft tissue point located at the inner commissure of each eye fissure*
	The crossing of the medial ends of the lower and upper eye lid curves
Nasion	*The mid‐point on the soft tissue contour of the base of the nasal root at the level of the frontonasal suture*
	The point where the brow ridge curves meet the superior extension of the mid‐line nasal profile curve
Pronasale	*The most anterior mid‐point of the nasal tip*
	The crossing of the mid‐line nasal profile and alar curves
Crista philtri	*The point at each crossing of the vermilion line and the elevated margin of the philtrum*
	The crossing of the upper lip and philtrum ridge curves
Labiale superius	*The mid‐point of the vermilion line of the upper lip*
	The crossing of the upper lip and mid‐line philtrum curves
Stomion	*The mid‐point of the horizontal labial fissure*
	The crossing of the mid‐line upper lip and labial seal curves
Labiale inferius	*The mid‐point of the vermilion line of the lower lip*
	The crossing of the lower lip and mid‐line lower lip curves
Gnathion	*The most anterior‐inferior mid‐point of the chin*
	The crossing of the mid‐line chin and mandible curves

The definitions in Tables 2 and 3 assume an image of a subject whose mouth is closed and whose eyes are open. In cases where stomion is affected by incomplete labial seal, this landmark will have to be split into upper and lower versions based on judgement of the point of contact with a complete labial seal. Of course, this issue applies to any definition of stomion. In cases where the eyes are closed, as commonly occurs with laser scan images, endocanthion and exocanthion are unavailable. An alternative is ektokonchion whose traditional definition is ‘the point of intersection of the lateral orbital margin and a transverse axis parallel with the upper orbital border, dividing the orbit in an upper and a lower half’. An adaptation to the new curve‐based definitions is ‘the crossing of the extensions of the inferior orbital and brow ridge curves’.

Although Tables 2 and 3 cover the majority of the principal facial landmarks, many others can be defined. One example is pogonion whose orientation‐based definition is ‘the most anterior mid‐point of the chin’. An adaptation to the new curve‐based definitions could be ‘the point of maximal curvature on the mid‐line chin curve’. However, it has to be recognised that in some subjects this can be a very flat region where a point of maximal curvature may be very difficult to identify. A role for definitions relative to other landmarks, for example ‘the point on the mid‐line chin curve that lies furthest from a line connecting sublabiale and gnathion’, may therefore remain. Similarly, flatness of the alar curve may make the identification of alare problematic, in which case it can be regarded as coincident with alare crest, which is indeed what happens using the traditional definitions.

**Figure 2:**
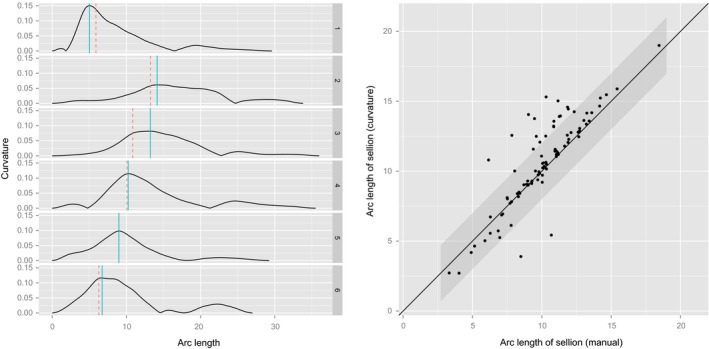
The left‐hand panel shows six examples of curvatures, each as a function of arc length, derived from the mid‐line nasal profile curve and its superior extension. The location of the dominant peak is indicated by the blue line and the location of sellion from the orientation‐based definition by the red dashed line. The right‐hand panel plots the locations of sellion from the orientation, and curvature‐based definitions on a sample of 91 subjects, with the dark grey shaded area indicating the region where the differences are < 2 mm.

It should be noted that the old and new definitions in Tables 2 and 3 do not necessarily correspond to the same anatomical locations. In many cases they are coincident, with the curve‐based definition aiming to provide a more robust definition, but in some cases, such as sellion, the orientation and curve‐based definitions may identify different points. There is no intrinsic difficulty with that as the two definitions have different but equally valid interpretations. However, it is worth checking that the curve‐based definitions are workable and effective. A validation study to address this is described in ‘Validation study’ but a small initial exploration based on sellion is reported here. This involved the numerical identification (using a computational algorithm) of the point of maximal curvature on the mid‐line nasal profile curve and its superior extension for 91 facial images. Each curve was represented in co‐ordinate form as *x*(*s*), *y*(*s*), *z*(*s*), where *s* is arc length as described at (1), using the method of p‐splines (Eilers & Marx, 1996), which includes smoothing, here using 8 degrees of freedom, to reduce the effect of surface noise. Six examples of the geodesic curvatures obtained from (1) are shown in the left hand side of Fig. 2. The presence of a dominant peak, indicated by the blue line, offers reassurance that a definition based on maximal curvature is well founded. The red dashed lines indicate the corresponding locations (along arc length) of sellion from manual identification using the traditional orientation‐based definition. In many cases these are very close, but in some cases there is a substantial difference. This is indicated for the entire sample in the plot of the arc length locations derived from the orientation and curvature definitions in the right hand panel of Fig. 2. This indicates a high concentration around the line of equality and a small proportion of substantial differences. Specifically, 70% of points lie with 1 mm and 79% of points within 2 mm (indicated by the grey shaded region). A further example of the definitions identifying different points is provided by gnathion, where the curve‐based definition usually corresponds to a location that is slightly lower than that based on orientation, as indicated in Fig. 1.

## Validation study

**Figure 3:**
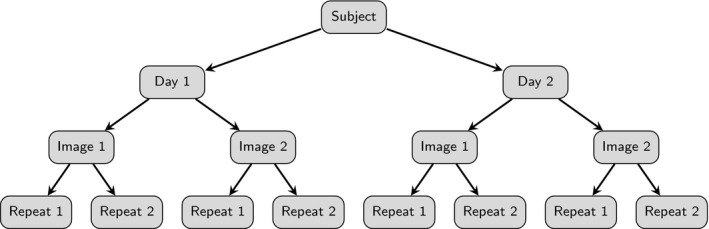
The hierarchical structure of the variability for a single observer of a single subject.

A new proposal for landmark definitions requires a validation study that will quantify the variation that operates when identification takes place in practice, and allow comparison of the orientation and curve‐based approaches. Several different levels at which variation is present were identified in Imaging perspective, and these need to be reflected in the study design. Figure 3 illustrates the structure for a single observer of a single subject, where each of four subjects were imaged twice on each of two different days. This allows the variation in facial shape between days and between repeats on the same day to be identified. Each of the four images was landmarked twice to identify the variation of repeat landmarking. All the images for each subject were landmarked by four different people, two of whom were trained on the orientation‐based definitions and two on the curvature‐based definitions.

As usual in the analysis of shape information, the collection of landmark configurations needs to be registered by Generalised Procrustes Analysis (gpa). As the repeat landmarks from the lowest level of the hierarchy refer to the same images, gpa was applied to the averages of the pairs lying at the lowest level of Fig. 3 and the translations, rotations and scaling for each average were applied to the individual configurations within these pairs to ensure that the whole collection was appropriately registered.

The hierarchical structure of the study design naturally leads to a multilevel model where random effects operate at each level. Pinheiro & Bates (2000) offer a comprehensive introduction to models of this type. If we consider the observation made by the observers applying the orientation‐based definitions, and denote by $v_{ijklm}$ the measurement recorded by observer *i* on subject *j* on day *k* with image capture *l* and repeat *m*, for a particular landmark in a particular dimension, then a natural model is

$$v_{ijklm}=\mu +o_{i}+s_{j}+d_{jk}+c_{jkl}+r_{jklm},$$

where μ denotes the mean value of the landmark co‐ordinate over the population and the random effects are represented by $o_{i}$, observer *i*; $s_{j}$, subject *j*; $d_{jk}$, day *k* for subject *j*; $c_{jkl}$, image capture *l* for day *k* for subject *j*; $r_{jklm}$, repeat *m* for image capture *l* for day *k* for subject *j*.

This allows an adjustment for each observer due to individual variation in the interpretation of the definitions, an adjustment for each subject because of changes in facial shape from person to person, as well as a nested set of adjustments to reflect the day/image/repeat hierarchy of measurements within each subject. All of these terms, apart from μ, are treated as random variables, each with its own associated standard deviation. These standard deviations are the parameters of interest as they express the size of the variation from each source. This type of model is well understood and is fitted here by maximum likelihood. As the aim of the exercise is to describe the levels of variation present, the model was fitted separately for the data from the observers applying the two different sets definitions. This allows any differential effects of the definitions to be expressed within any of the random effects involved.

**Figure 4:**
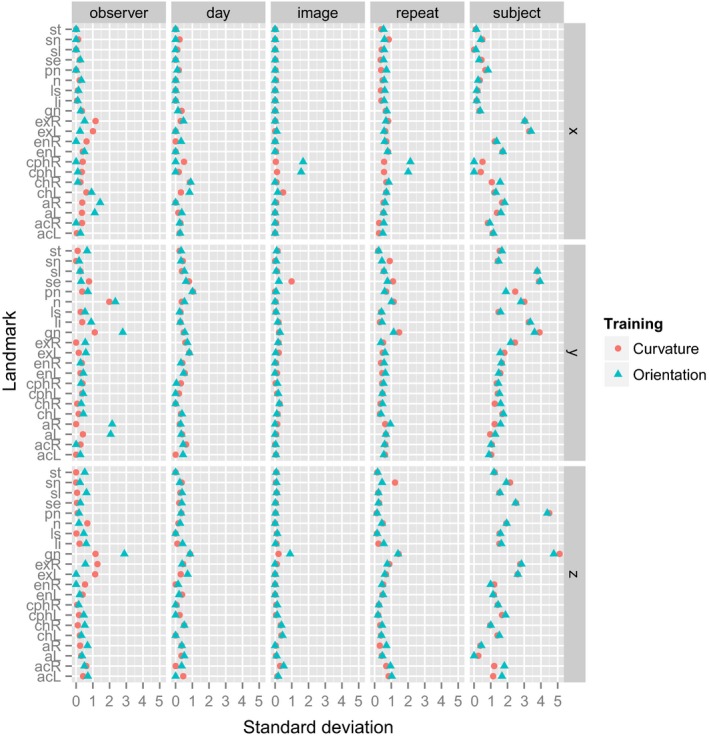
Estimated standard deviations at different levels of variation in identified landmark locations in *x*,* y* and *z* directions (mm), separated by the use of orientation‐ and curvature‐based definitions.

Figure 4 displays the estimated standard deviations for all the random effects, separately for each landmark in each dimension, and with the definitions used identified by symbol and colour. The panels in each column show the results for the *x*,* y* and *z* dimensions, respectively, where *x* refers to left‐right, *y* to bottom‐top and *z* to back‐front, based on orientation of the Procrustes mean shape to place the mean values of the exocanthions along the horizontal axis, and the mean values of nasion and subnasale vertically. This simply allows a clear interpretation of the *x*,* y*,* z* co‐ordinates. The subject variation is not of primary interest because we know that there is substantial natural variation in facial shape among people. The day and image variations are very small as expected. The variation in repeat identification is modest, with little evidence of systematic differences between the definition groups. (The results for the christa philtrum landmarks are an exception at image and repeat levels, with high variation in the *x* co‐ordinate.) The observer variation is interesting as this quantifies the extent to which different observers place the landmarks in different locations. In the case of nasion there is a high level of variation for both definitions, which is not surprising because of the intrinsic difficulty in locating this landmark on a relatively flat region. Interestingly, the curvature definition group shows substantial improvement in variation for gnathion and alare and modest improvement for several other landmarks. Table 4 reports the random effect standard deviations averaged across landmarks and dimensions, as an overall summary. The reduction in variation at observer level for the curvature group is marked, from 0.553 to 0.361. With a very small number of observers involved in the validation study, this cannot be taken as conclusive evidence of a superiority of the curvature‐based definitions. It does, however, offer encouragement for the use of this approach.

**Table 4:** Standard deviation (mm) of random effects, averaged over all landmarks and dimensions

	Curvature	Orientation
Observer	0.361	0.553
Day	0.288	0.323
Image	0.103	0.086
Repeat	0.562	0.587
Subject	1.645	1.670

A second small study was carried out for confirmation. Twenty observers were trained, 10 each on the curvature and orientation methods, with the larger number of observers allowing stronger focus on this key effect. The observers were asked to locate the difficult landmark gnathion on five subjects, each with two repeat images, but the landmarks alare crest L/R and crista philtrum L/R were also located in order to allow Procrustes registration. The curvature method showed a reduction of 13% in observer variability in the *y* co‐ordinate, where location of gnathion is most problematic.

**Figure 5:**
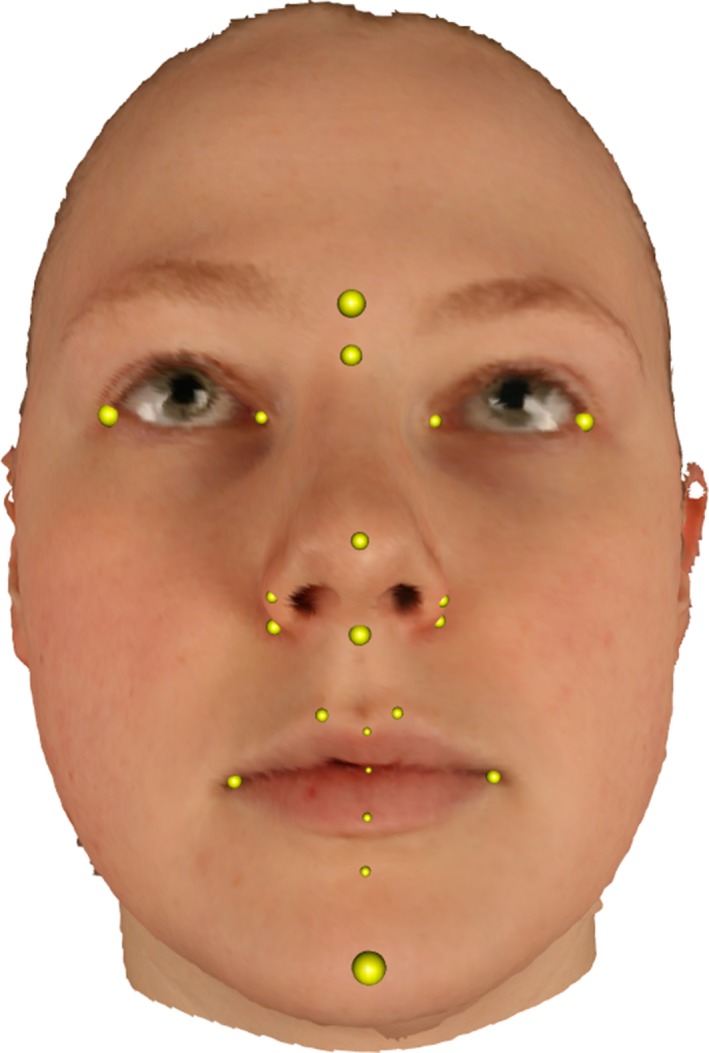
Facial image with spheres whose radii are proportional to the reproducibility of each landmark, using curvature‐based definitions.

For a single landmark identification on one individual, the relevant measure of reproducibility combines the variability at all the levels shown in Table 4 apart from subject. Under a working assumption of independence of the effects across dimensions, simply as a means of providing a useful summary measure, the average standard deviations across the landmarks are 1.27 and 1.51 for the curvature and orientation approaches, respectively. Figure 5 shows a facial image with spheres whose radii are proportional to the standard deviations associated with each landmark, using curvature‐based definitions, to give a more informative picture of the reproducibility

## Discussion

The principal aim of this paper has been to propose an approach to defining facial landmarks that avoids the need for careful orientation and that properly exploits the three‐dimensional nature of the images that are now routinely available. Methods of quantifying and classifying three‐dimensional surface shape have provided the key and, in particular, anatomical curves have proved to give a very useful basis for the identification of landmarks. This has the appeal of exploiting the familiar large‐scale structure of the face rather than basing definitions only on the immediate neighbourhoods of point locations. The majority of standard landmarks can be defined as points of maximal geodesic curvature or as the crossing points of two or more anatomical curves. A validation study, while can be regarded only as indicative because of the small number of observers involved, has nonetheless provided some supporting evidence for improvement in reproducibility through use of the curve‐based definitions.

The availability of quantifiable measures of surface shape leads naturally to the question of the extent to which good definitions of landmarks might lead to helpful methods of automatic, rather than manual, identification. There is a considerable body of work on this topic; see, for example, Çeliktutan et al. (2013). Some methods aim at exploiting simple characteristics of the surface, such as curvature (Pamplona Segundo et al. 2010) or projections into particular orientations (Peng et al. 2011), combined with some prior knowledge about the geometry of the human face. For example, the nose and chin tips can be considered as ‘peaks’ or ‘caps’ with characteristic curvature indices, while the eye and mouth corners are ‘pits’ or ‘cups’. The prior knowledge is often provided as a set of empirical rules that have been found to achieve satisfactory performance. An outstanding example is the work by Gupta et al. (2010), who derived rules based on statistics from anthropometric studies. These approaches have an attractive directness and simplicity, once suitable curvature characteristics have been constructed.

In contrast to the above, other methods use the information in sets of previously identified landmarks, and their relative configurations, to give guidance on the positions of the landmarks that might reasonably be expected in a new facial image. This approach seeks to build a statistical or ‘machine learning’ model that could be considered as an analogue of the ways in which the human brain exploits previous experience in making judgements about the plausibility of appropriate positions on a new face. Popular examples of this strategy include statistical shape models (Perakis et al. 2013; Sukno et al. 2015) and graph matching (Jahanbin et al. 2008). The construction of appropriate training sets from which to derive prior distributions for landmark locations can prove challenging and is a crucial element of these methods, as the accuracy of these algorithms can be highly sensitive to the quality of the training landmarks. However, this approach has the advantage of considerable flexibility, as the rules for locating the landmarks of interest are implicitly derived in an automatic manner. As a consequence, these methods are usually able to target larger subsets of landmarks and cope with arbitrary definitions of the points, as long as they are consistent with the annotations provided.

In terms of performance of automatic landmarking methods, the best results reported to date indicate average errors between 1.5 and 3.5 mm for the most distinctive facial landmarks. Sukno et al. (2015) is an example of recent work in this area.

However, the role of anatomical curves in the landmark definitions discussed within this paper also raises the prospect of using these to characterise faces in a much richer way than individual point locations. For example, the highlighted difficulty in defining and identifying gnathion may be circumvented by analysis of the entire underlying mid‐line curve that expresses the shape of the whole region. With suitable methods of statistical analysis this can provide a rich description of the local anatomy without the need to identify a single point as a definitive landmark.
